# Prevalence of carbapenem-resistant and extended-spectrum beta-lactamase-producing Enterobacteriaceae in a teaching hospital in Ghana

**DOI:** 10.1371/journal.pone.0274156

**Published:** 2023-10-30

**Authors:** James Sampah, Isaac Owusu-Frimpong, Frank Twum Aboagye, Alex Owusu-Ofori

**Affiliations:** 1 Department of Clinical Microbiology, School of Medicine and Dentistry, Kwame Nkrumah University of Science and Technology, Kumasi, Ghana; 2 Laboratory Department, St. Patrick’s Hospital, Offinso, Ghana; 3 Department of Molecular Microbiology and Immunology, Bloomberg School of Public Health, Johns Hopkins University, Baltimore, Maryland, United States of America; 4 CSIR-Water Research Institute, Biomedical and Public Health Research Unit, Accra, Ghana; 5 Clinical Microbiology Unit, Laboratory Services Directorate Komfo Anokye Teaching Hospital, Kumasi, Ghana; Tribhuvan University, NEPAL

## Abstract

**Background:**

Carbapenem-resistant Enterobacteriaceae (CRE) and Extended-spectrum beta-lactamase (ESBL) production among Gram-negative Enterobacteriaceae is an increasing global challenge due to the high morbidity and mortality associated with their infections, especially in developing countries where there are little antibiotic treatment options. Despite these challenges, few studies in Ghana have described the burden of CRE. Therefore, this study aimed to determine the prevalence of carbapenem-resistant Enterobacteriaceae isolated from patients at the Cape Coast Teaching Hospital (CCTH) in the Central region of Ghana.

**Methodology/Principal findings:**

Enterobacteriaceae isolates were collected from April to July 2019 at the bacteriology unit of CCTH using a consecutive sampling method. Isolates were identified by standard microbiological techniques and confirmed using API 20E. Kirby Bauer disc diffusion method was used to determine the antibiogram of isolates. Isolates were also subjected to ESBL testing using the single-disc combination method. Carbapenem-resistant isolates were identified by the Kirby Bauer disc diffusion method and then examined genotypically for the presence of *blaKPC-1*, *blaIMP-1*, *blaVIM-1*, *blaNDM-1*, and *blaOXA-48* genes via polymerase chain reaction (PCR). Of the 230 isolates comprising *E*. *coli* (40.9%), *Citrobacter spp*. (32.6%), *K*. *pneumoniae* (9.1%), *P*. *mirabilis* (6.1%), *P*. *vulgaris* (5.2%), *Enterobacter spp* (3.5%)., *K*. *oxytoca* (2.2%), and *Serratia marcenses* (0.4%). Most isolates were from urine 162(70.4%) and wound samples. The isolates showed high resistance to ampicillin 171 (74.3%) and cefuroxime 134(58.3%). The prevalence of MDR was 35.2% (81), with *E*. *coli* 40(42.6%) being the majority that exhibited MDR. Of the 230 isolates, 113(49.1%) were ESBL producers, with *E*. *coli* 54(57.5%) accounting for the majority, while *Serratia marcenses* was the least. Of the 13 (5.7%) CRE isolates that showed resistance towards carbapenem in the disc diffusion method, 11 showed the presence of the *blaNDM-1* gene, while all isolates showed the presence of the *blaOXA-48* gene.

**Conclusion:**

The prevalence of carbapenem resistance and ESBL-producing Enterobacteriaceae pathogens among patients at the Cape Coast Teaching Hospital is high and alarming. Therefore, it is imperative to consider effective infection prevention and control measures should be implemented at the hospital to prevent the rapid spread of these dangerous organisms.

## Introduction

The occurrence of antimicrobial resistance (AMR) is a significant challenge in the treatment of infections, and these infectious diseases are ever-increasing, especially with the re-emergence of pathogens [1]. AMR occurs naturally and is known to be escalated by the misuse and overuse of antimicrobial agents [2]. Therefore, the increasing emergence of multidrug-resistant (MDR) Gram-negative bacilli is of significant priority in clinical settings across the globe. Managing Gram-negative multidrug-resistant (MDR) infections has become significantly challenging over the past two decades in many developing countries, especially in the Sub-Saharan region [3]. These infections are usually associated with high morbidity rates, high mortalities, and extended hospital stays [4].

Enterobacteriaceae account for more than 30% of bacterial infections with high morbidity and mortality outcomes [5,6]. Meningitis, urinary tract infections, gastroenteritis, septicemia, pneumonia, and wound infections are just a few of the conditions that these organisms commonly cause [7,8]. Enterobacteriaceae are well known for their global public health threat due to their increasing antimicrobial resistance. Studies have established that members of this family of bacteria gain their antimicrobial resistance via the acquisition of drug-resistance genes through mobile genetic elements such as transposons and plasmids transferred within the same species or different species [9,10]. The acquired resistance genes facilitate the production of β-lactamase enzymes, especially the extended-spectrum β-lactamase (ESBL), responsible for conferring resistance to most β-lactam antibiotics [7,10–13]. For instance, carbapenems, a class of β-lactam antibiotics, have been established to lose their potency against Enterobacteriaceae due to resistance [7,14]. Furthermore, other well-known antimicrobials such as fluoroquinolones, aminoglycosides, phenicols, sulfonamides, and tetracyclines have been rendered ineffective by this group of bacteria, thereby making treatment of infections of these bacteria difficult [15–17].

Carbapenems are broad-spectrum β-lactam antibiotics globally regarded as the ‘last-line’ antibiotics; thus, they are considered the last choice of drug for the treatment of critically ill patients and/or those infected with resistant Gram-negative bacteria [18]. They are essentially reserved for cases of suspected MDR bacterial infections. This class of β-lactam is very similar to penicillin and cephalosporin [19]. Carbapenems are bactericidal in their mode of activity against Gram-negative bacterial species. However, unlike other β-lactam antibiotics, carbapenems invade the bacterial cell through the outer membrane proteins (*OprDs*), other than those used by the cephalosporin and penicillin (*OmpC* and *OmpF*), which results in the interruption of cell wall formation [18]. Once the cell wall formation is interrupted by the carbapenems, the peptidoglycan layer becomes very weak, and the cell eventually bursts, leading to the death of the bacterial cell [20,21].

Generally, carbapenem-resistant Enterobacteriaceae (CRE) has been studied and reported in a few African countries such as Tanzania, South Africa, Nigeria, Kenya, Morocco, and Ghana [14,22–36]. In Ghana, reported carbapenem resistance is not too different from other developing and under-developed African countries. In a study by Codjoe and colleagues among Gram-negative isolates collected from four hospitals in Ghana, a 2.9% prevalence of carbapenem-resistant was reported [37]. The report further indicated that 23.4% of the bacterial isolates harbored known carbapenem-resistant genes; *blaVIM-1*, *blaOxa-48*, and *blaNDM-1*. Another study conducted by Quansah and colleagues reported carbapenem-resistant genes *OXA- 48* (2.16%) and *NDM-1* (0.72%) in the study population [35].

While a few studies in other parts of Ghana have looked at carbapenem resistance in Enterobacteriaceae, there are no published data on CRE from the Central Region of Ghana. As a result, this study aimed to determine the prevalence of carbapenem resistance, MDR, and ESBL-producing Enterobacteriaceae in the Cape Coast Teaching Hospital in the Central Region of Ghana.

## Materials and methods

### Ethics statement

Cape Coast Teaching Hospital Ethical Review Committee (CCTHERC/EC/2019/044) of the Cape Coast Teaching Hospital and Committee on Human Research, Publication and Ethics (CHRPE/AP/201/19) of the School of Medical Sciences, Kwame Nkrumah University of Science and Technology jointly approved the study. Written consent was obtained from all participants and signed either by thumbprint or signature after an explanation of the procedure and the purpose of the study was provided to the patient. In addition, written consent was obtained from parents or guardians for participants below 18yrs.

### Study setting

**Fig 1:**
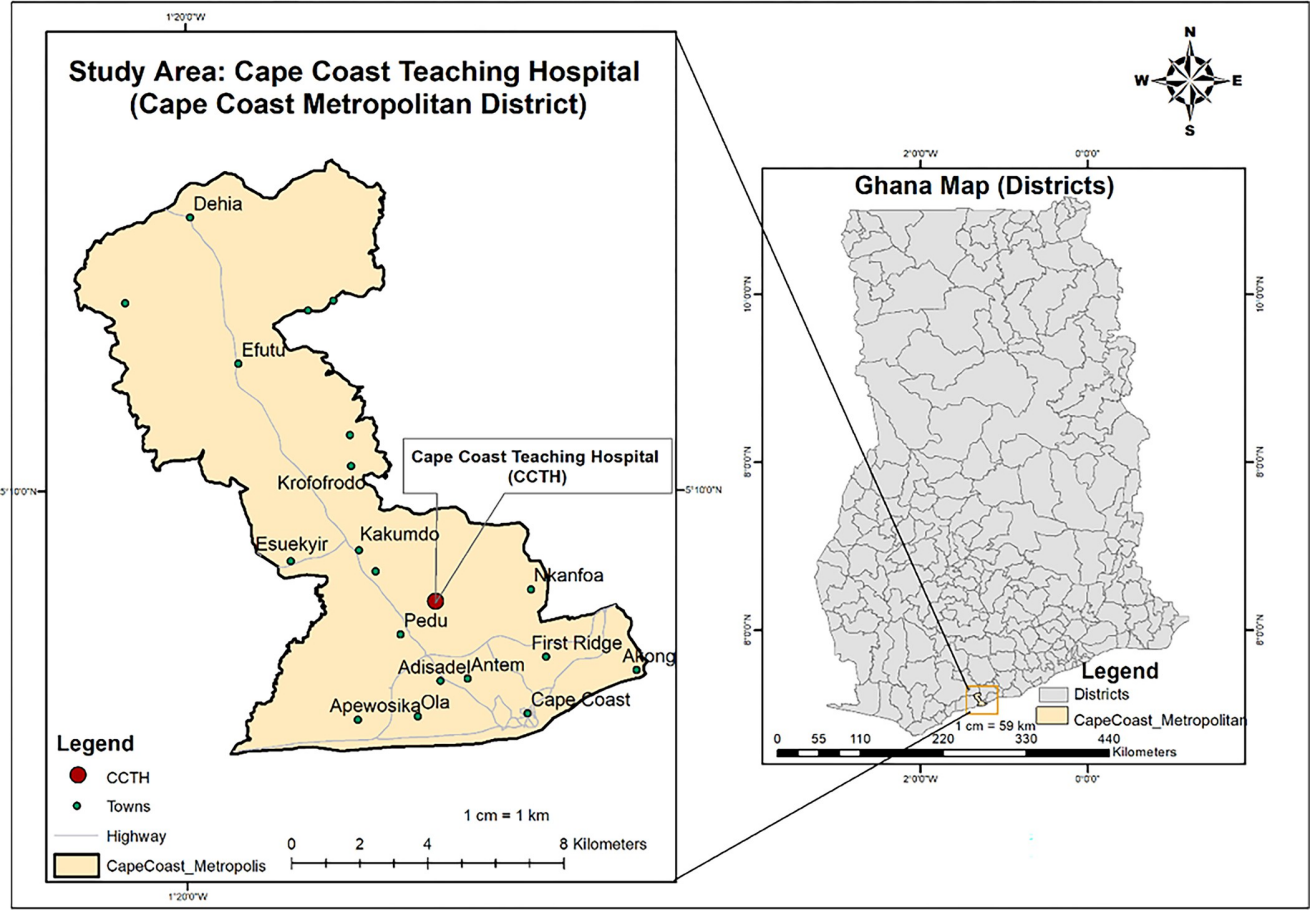
The map of Ghana showing the location of Cape Coast Teaching Hospital in the Cape Coast Metropolitan District. *Map developed with ESRI ArcMap 10*.*8 using data from Ghana Open Data Initiative*, *and OpenStreetMap and OpenStreetMap Foundation*.

The study was conducted between April and October 2019 at Cape Coast Teaching Hospital (CCTH) in Cape Coast in the Cape Coast Metropolitan, in the Central region of Ghana (Fig 1). The health facility is a tertiary government healthcare facility with a 420-bed capacity. In 2018, the facility recorded outpatient attendance of 158,164, while the total admission was 10,865 [38]. During the period of sample collection (April to July 2019), the diagnostic bacteriology unit received and processed 1,388 clinical specimens [38]. CCTH serves as a referral hospital for the Central Region and other parts of the Western Region. The metropolitan has a recorded population of 169,894, which comprises 82,810 (48.7%) males and 87,084 (51.3%) females [39].

### Bacterial collection

Two hundred and thirty (230) non-repetitive Enterobacteriaceae were isolated and identified from the clinical specimens submitted using standard bacteriological techniques and a panel of biochemical tests. Clinical specimens used include urine, blood, sputum, high vaginal swab (HVS), endocervical swab, wound swab, stool, and semen. In addition, using a pretested questionnaire, socio-demographic data, including age and sex, were recorded. Isolates identification was confirmed using the API 20E strip (BioMerieux, France). Isolates were maintained in 20% brain heart infusion glycerol broth at -20°C for further testing.

### Antibiotic susceptibility testing

All isolates from glycerol broth were subcultured on MacConkey agar media after partially thawing the broth. Antibiotic susceptibility testing (AST) was performed on Mueller Hinton agar using the Kirby Bauer disc diffusion method and interpreted according to the Clinical and Laboratory Standards Institute (CLSI) guideline [40]. The AST was performed using the following antibiotic discs; Ampicillin (10μg), Ciprofloxacin (5μg), Levofloxacin (5μg), Ofloxacin (5μg), Gentamicin (10μg), Amikacin (30μg), Ceftriaxone (30μg), Ceftazidime (30μg), Cefotaxime (30μg) and Cefuroxime (30μg) which were from Biomark Laboratories, India. In addition, antibiotic discs Meropenem (10μg), Imipenem (10μg), and Ertapenem (10μg) from Oxoid, UK, were included. *E*. *coli* (NCTC 19418) was used as a quality control strain. The zones of inhibitions were recorded and interpreted according to the Clinical and Laboratories Standards Institute guidelines [40]. Multidrug resistance was defined as resistance to at least three classes of antibiotics [20].

### Phenotypic screening and confirmation for ESBL

ESBL detection was performed on Mueller Hinton agar seeded with the test organism. All 230 isolates were screened for ESBL-producing enzymes using Ceftazidime (30μg) and Cefotaxime (30μg) discs according to the method described by the CLSI guideline [40]. An isolate resistant to any of the screening antibiotic discs was suspected of ESBL and reported as positive for ESBL screening. ESBL confirmation was done using a single disc of Oxoid Cefpodoxime (10μg) alone and Cefpodoxime/ clavulanic acid (10/1μg) using the Kirby Bauer disc diffusion method [40]. The confirmatory discs were placed on the seeded Mueller Hinton agar, ensuring that the discs were at least 20mm apart and incubated at 37°C overnight. An enhanced zone of inhibition (≥ 5 mm) around the Cefpodoxime/ clavulanic acid (10/1μg) relative to the single disc of Cefpodoxime (10μg) was considered positive for the production of ESBL. *Escherichia Coli* ATCC 25299 and *K*. *pneumoniae* ATCC 700603 were used as quality controls.

### Molecular detection of carbapenem-resistant genes

After the antibiotic susceptibility testing using antibiotic discs, resistant and intermediate susceptible bacterial isolates were subjected to molecular confirmation with polymerase chain reaction (PCR) to detect *blaKPC-1*, *blaIMP-1*, *blaVIM-1*, *blaNDM-1*, and *blaOXA-48* genes.

#### Bacterial DNA extraction

The DNA of bacterial isolates was extracted using Quick DNA kits (Zymo Research, USA) according to the manufacturer’s procedure. With a sterile loop, a loopful of the isolates were picked from the Mueller Hinton agar plate and emulsified in sterile 1.5 mL microcentrifuge tubes containing 400 μL of Genomic Lysis Buffer and 5 μL Proteinase K. These tubes were vortexed at 2500 rpm for 30sec and incubated at 56°C overnight. After the overnight incubation, mixtures were vortexed again, transferred to Zymo-Spin™ IIC Columns in collection tubes, and centrifuged at 10,000 x g for one minute. The flowthroughs were discarded, after which 200 μL of DNA Pre-Wash Buffer was added to each spin column. Centrifugation was performed again at 10,000 x g for one minute. Afterward, the final washing was done by adding 500 μL of g-DNA Wash Buffer to the spin column and centrifuging again at 10,000 x g for one minute. Finally, the spin columns were transferred into sterile 1.5 mL microcentrifuge tubes, after which 100 μL of Elution Buffer was added to elute the DNA. DNA samples were stored at -20°C before proceeding to downstream analysis. Prior to the PCR, Bacterial DNA quantification was performed with the Qubit 3.0 fluorometer (Life Technologies Holdings Pte Ltd, Malaysia), and the values were recorded.

#### Detection of carbapenem resistance-encoding genes using PCR

Carbapenem-resistant isolates were genotypically examined for *blaKPC-1*, *blaIMP-1*, *blaVIM-1*, *blaNDM-1*, and *blaOXA-48* genes by PCR methods using gene-specific primers (Table 1) described by Poirel and colleagues [41]. The PCR assay was carried out with a 20 μL reaction mixture containing 2 μL genomic-DNA, 1x Standard reaction buffer, 0.3mM each of dATP, dCTP, dTTP, and dGTP, 200 nM each of Forward and Reverse primers, and 1.25 U One*Taq* DNA Polymerase (New England Biolabs Inc., USA). PCR reactions were performed using the Bio-Rad PTC-200 Thermal Cycler (Bio-Rad Laboratories, USA) with the following cycling conditions; an initial denaturation at 94°C for 3 mins, followed by 40 cycles at 94°C for 30sec, 56°C for 30sec, and 68°C for 30sec. Finally, an elongation step was performed at 68°C for 5 minutes. Afterward, the PCR products were resolved by agarose gel electrophoresis and visualized under UV light using UVP Bio-Doc- It Imaging system–trans-illuminator (AnalytikJena, Germany).

**Table 1:** Carbapenem-resistant genes primer sets.

Gene	Sequence 5’ 3’	Fragment size (bp)
*blaKPC-1*	**Forward**: CGTCTAGTTCTGCTGTCTTG **Reverse**: CTTGTCATCCTTGTTAGGCG	798
*blaIMP-1*	**Forward**: GGAATAGAGTGGCTTAAYTCTC **Reverse**: GGTTTAAYAAAACAACCACC	232
*blaVIM-1*	**Forward**: GATGGTGTTTGGTCGCATA **Reverse**: CGAATGCGCAGCACCAG	390
*blaNDM-1*	**Forward**: GGTTTGGCGATCTGGTTTTC **Reverse**: CGGAATGGCTCATCACGATC	621
*blaOXA-48*	**Forward**: GCGTGGTTAAGGATGAACAC **Reverse**: CATCAAGTTCAACCCAACCG	438

**NB**: KPC, *Klebsiella pneumoniae* carbapenemase; IMP, Imipenemase; VIM, Verona integron-encoded metallo-β-lactamase; NDM, New Delhi metallo-β-lactamase; OXA-48, oxacillinase-48.

### Statistical analysis

Data were entered using Microsoft Excel 2019 and analyzed using GraphPad Prism version 8.0 (Graphpad Inc., La Jolla, CA, USA). A simple frequency was used to describe the study population with the socio-demographic and other relevant variables.

## Results

### Distribution of clinical specimen

**Fig 2:**
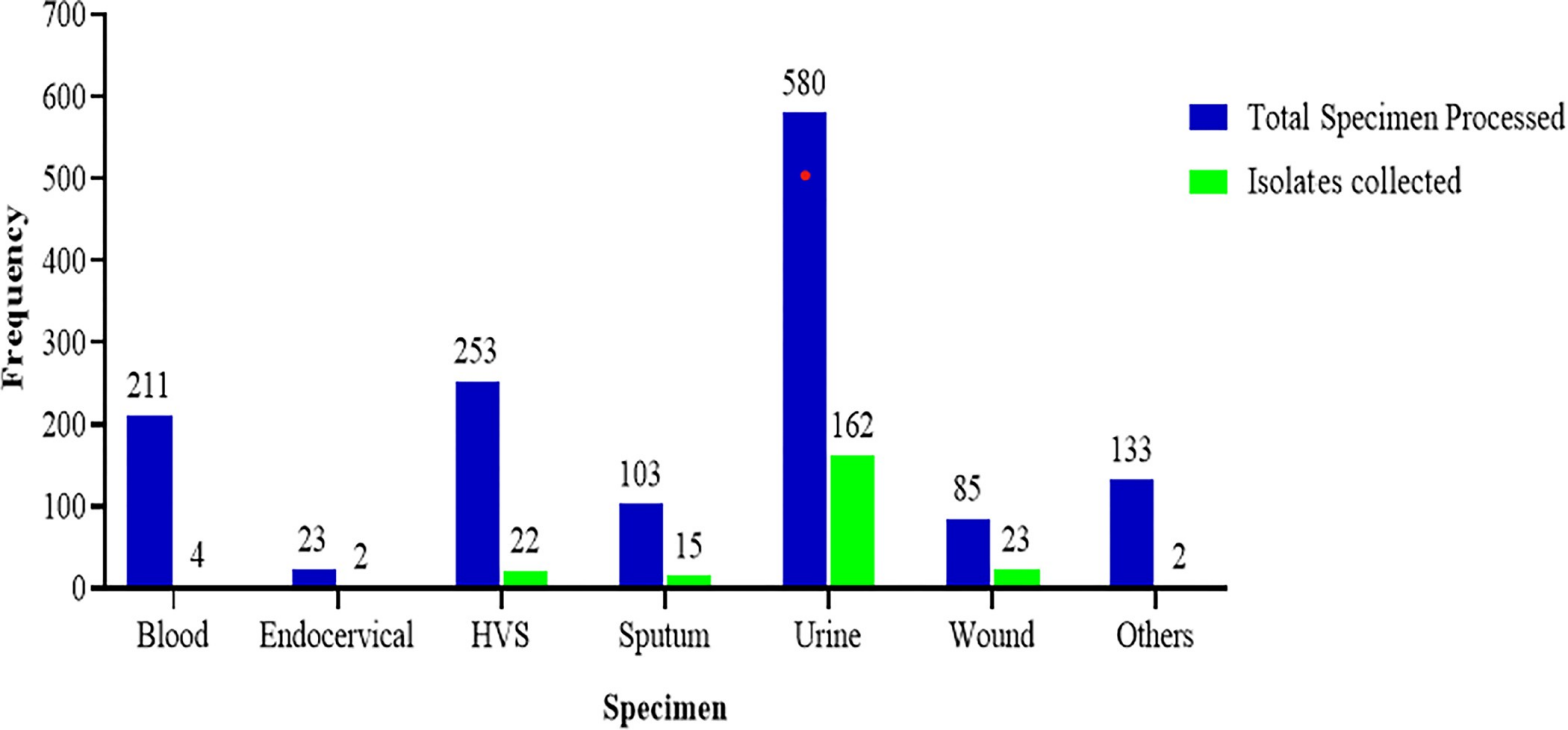
Distribution of samples received, and isolates collected.

Out of the 1388 samples processed, 230 isolates were identified as Enterobacteriaceae; 162 (70.4%) isolates were from urine, 23 (10.0%) from wound swabs, 22 (9.6%) from HVS, 15 (6.5%) from sputum, 4 (1.7%) from blood, 2 (0.8%) from the endocervical swab, 1 (0.4%) from other types of specimens (Fig 2).

### Distribution of isolates/organism

Of the 230 isolates collected for this study, *Escherichia coli* was the most frequent pathogen, 94 (40.9%), followed by *Citrobacter* spp. 75 (32.6%), *Klebsiella pneumoniae* 21 (9.1%), *Proteus vulgaris* 12 (5.2%), *Proteus mirabilis* 14 (6.1%), *Enterobacter* spp. 8 (3.5%), *Klebsiella oxytoca* 5 (2.2%) and *Serratia marcenses* 1 (0.4%) (Table 2).

**Table 2:** Distribution of isolates.

	Point of Care		
Isolate	Outpatient [N]	In-patient [N]	Total [N] (%)
***E*. *coli***	80	14	94 (40.9)
***Citrobacter* spp.**	66	9	75 (32.6)
***K*. *pneumoniae***	19	2	21 (9.1)
***P*. *vulgaris***	9	3	12 (5.2)
***P*. *mirabilis***	8	6	14 (6.1)
***Enterobacter* spp.**	8	0	8 (3.5)
***K*. *oxytoca***	5	0	5 (2.2)
***Serratia marcenses***	1	0	1 (0.4)
**Frequency**	**196**	**34**	**230 (100)**

### Antibiotics susceptibility pattern of Enterobacteriaceae

**Fig 3:**
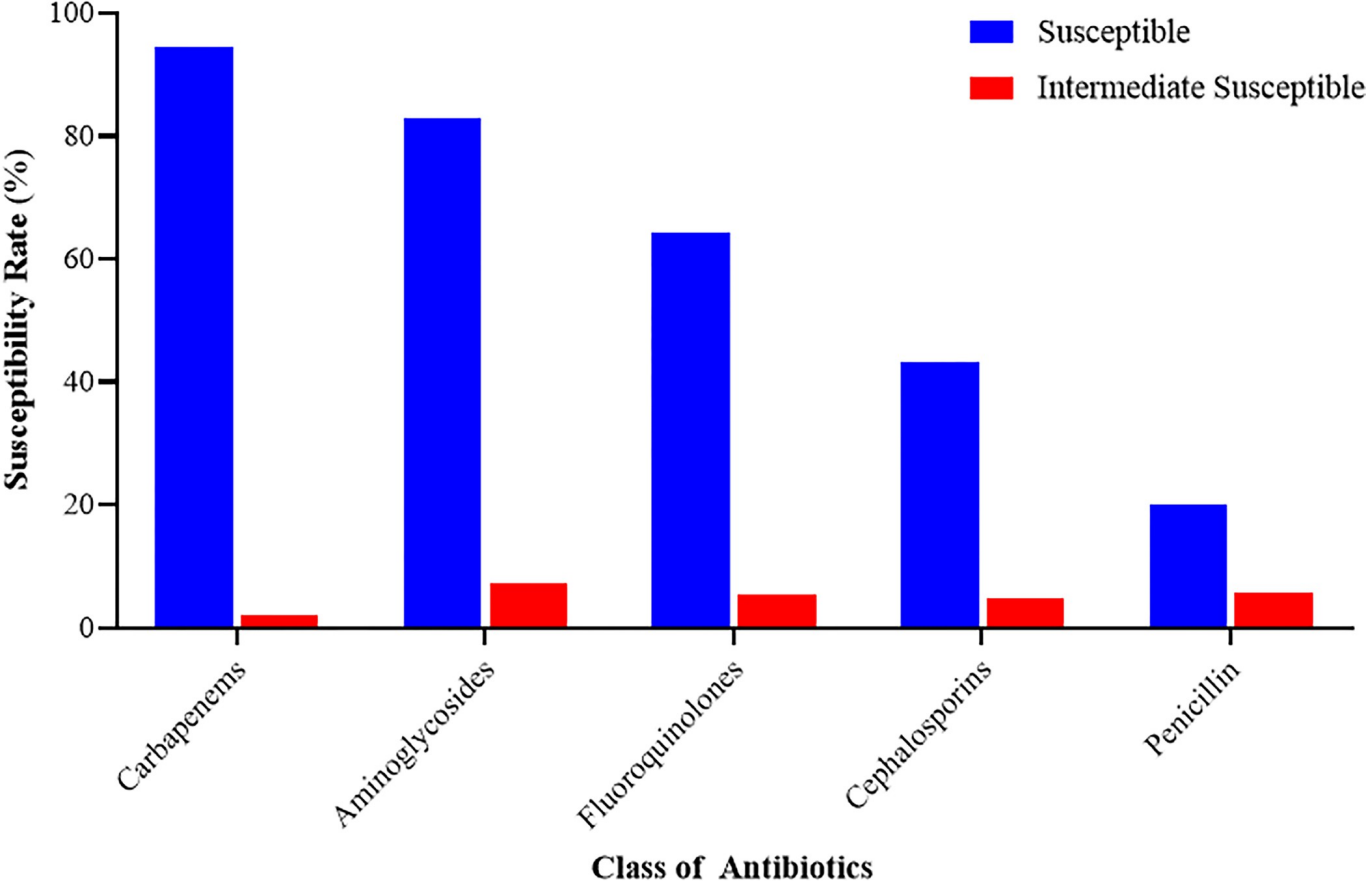
Susceptibility of isolates to the different classes of antibiotics.

Of the 13 antibiotics tested, susceptibility was highest to amikacin (AMK) (97.8%), followed by meropenem (MEM), imipenem (IMI), and ertapenem (ERT) with frequencies of 224 (97.4%), 224 (97.4%), and 204 (88.7%), respectively. The antibiotics to which isolates were most resistant were ampicillin (AMP) (74.4%), cefuroxime (CXM) (58.3%), and cefotaxime (CTX) (50.9%). Concerning the use of other antibiotics, isolates were resistant to Ciprofloxacin (33.0%) and gentamicin (19.2%) (Table 3). Penicillin (74.3%) was the class of antibiotic to which isolates showed the most resistance, while carbapenem (3.6%) was the class of antibiotic to which isolates showed the least resistance (Fig 3).

**Table 3:** Antibiotic susceptibility profile of Enterobacteriaceae.

Antibiotic	Susceptible *n* (%)	Intermediate *n* (%)	Resistant *n* (%)
Ampicillin	46 (20)	13 (5.7)	171 (74.3)
Gentamicin	156 (67.8)	30 (13.0)	44 (19.1)
Amikacin	225 (97.8)	3 (1.3)	2 (0.9)
Ciprofloxacin	141 (61.3)	13 (5.7)	76 (33.0)
Levofloxacin	158 (68.7)	12 (5.2)	60 (26.1)
Ofloxacin	145 (63)	13 (5.7)	72 (31.3)
Cefuroxime	80 (34.8)	16 (6.9)	134 (58.3)
Cefotaxime	104 (45.2)	9 (3.9)	115 (50.9)
Ceftazidime	106 (46.1)	9 (3.9)	115 (50.0)
Ceftriaxone	108 (47.0)	9 (3.9)	113 (49.1)
Ertapenem	204 (88.6)	13 (5.7)	13 (5.7)
Meropenem	224 (97.4)	0 (0.0)	6 (2.6)
Imipenem	224 (97.4)	0 (0.0)	6 (2.6)

### MDR and Extended-Spectrum Beta-Lactamase (ESBL) producing organisms

Out of the 230 isolates collected, eighty-one (81) isolates exhibited multidrug resistance (35.2%) toward antibiotics used. Among isolates that showed MDR, *E*. *coli* (42.6%) was the majority, while *Serratia marcenses* was the least. Of 230 isolates screened for ESBL production, 113 (49.1%) were ESBL producers, while 117 (50.9%) were non-ESBL producers. Among the ESBL producers, *E*. *coli* (23.5%) was the majority, while *Serratia marcenses* (0.4%) was the least. ESBL producers were found among all the species of isolates collected. (Table 4).

**Table 4:** MDR and ESBL among isolates.

Bacterial isolates	Number of isolates (N)	MDR n (%)	ESBL n (%)	Non-ESBL n (%)
*E*. *coli*	94	40 (42.6)	54 (57.5)	40 (42.6)
*Citrobacter* spp.	75	25 (33.3)	31 (41.3)	44 (58.7)
*Klebsiella pneumoniae*	21	5 (23.8)	10 (47.6)	11 (52.4)
*Proteus mirabilis*	14	4 (28.6)	7 (50.0)	7 (50.0)
*Proteus vulgaris*	12	3 (25.0)	4 (33.3)	8 (66.7)
*Enterobacter* spp.	8	2 (25.0)	3 (37.5)	5 (62.5)
*Klebsiella oxytoca*	5	1 (20.0)	3 (60)	2 (40.0)
*Serratia marcenses*	1	1 (100)	1 (100)	0 (0.0)
**Frequency**	**230**	**81 (35.2)**	**113 (49.1)**	**117 (50.9)**

### Molecular detection of Carbapenem-resistant genes

**Fig 4:**
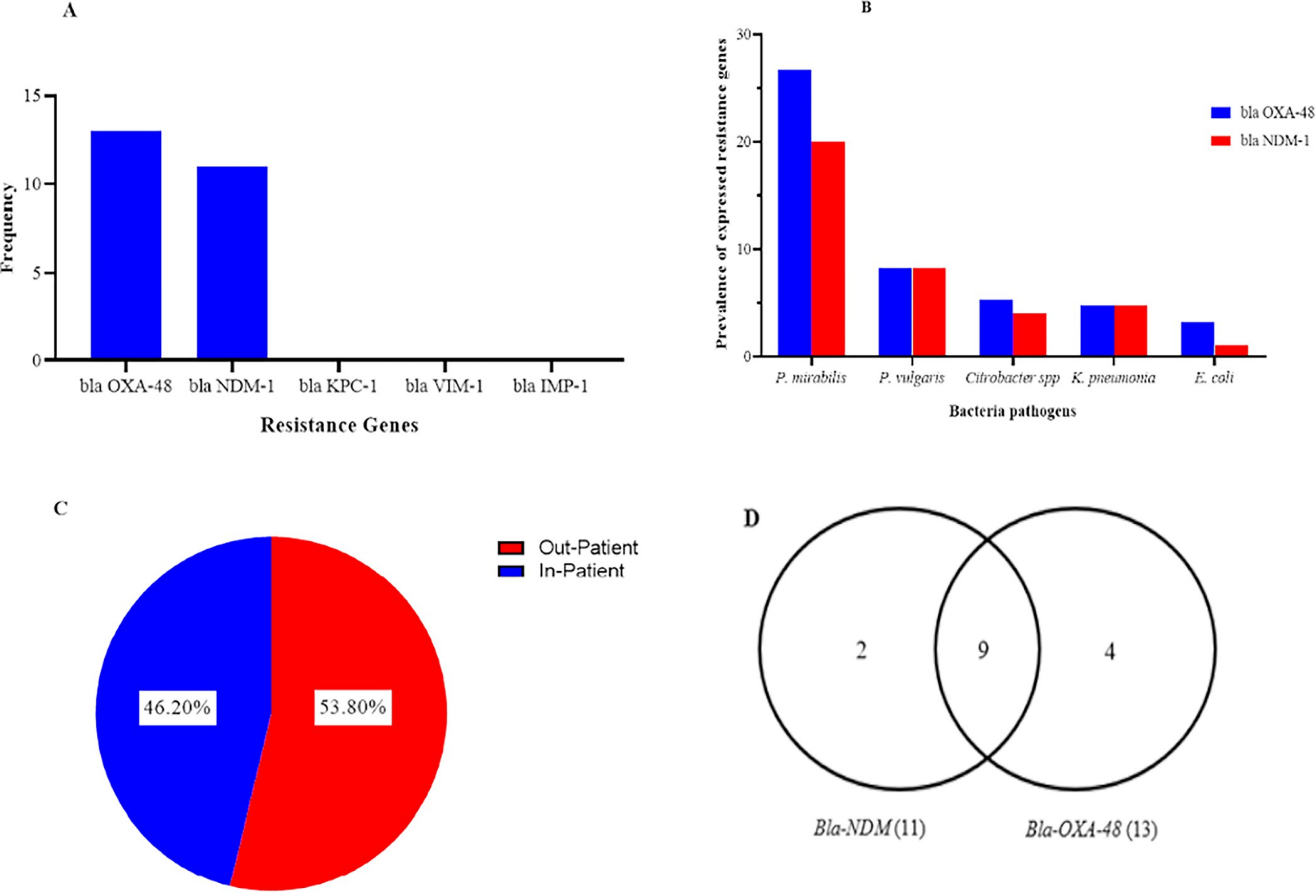
The outcome of Carbapenem-resistant genes from the molecular detection showing the presence of Carbapenem-resistant gene (A), distribution of *blaOXA-48* and *blaNDM-1* genes among CRE isolates (B), location of patients with CRE (C), and the distribution of *blaOXA-48* and *blaNDM-1* genes (D).

Thirteen (13) isolates that showed resistance to at least one of the three carbapenems and the 13 isolates that showed intermediate resistance to only ertapenem from the Kirby Bauer disc diffusion test were selected for PCR (Fig 4A). None of the isolates selected for PCR showed the presence of *blaKPC-1*, *IMP-1*, and *VIM-1* genes. However, *blaOXA-48* and *blaNDM-1* were detected among some of the Carbapenem-Resistant Enterobacteriaceae (CRE) isolates (Fig 4A). Of the 13 resistant isolates, 11 showed the presence of the *blaNDM-1* gene, while all resistant CRE showed the presence of the *blaOXA-48* gene (Fig 4D). Also, 9 of the 13 resistant isolates exhibited both *blaOXA-48* and *blaNDM-1* resistant genes. *P*. *mirabilis* (26.7%) was the Enterobacteriaceae which exhibited more of the *OXA-48* gene, while *E*. *coli* (3.2%) was the least (Fig 4B). Similarly, *P*. *mirabilis* (20.0%) showed more of the presence of *NDM-1* gene, while *E*. *coli* (1.1%) was the least (Fig 4B). The 13 resistant isolates with detected carbapenem-resistant genes were found among outpatients (53.8%) and in-patients (46.2%). Finally, the *blaOXA-48* gene was also found among all 13 intermediate susceptible isolates (Fig 4C).

## Discussion

The knowledge of the distribution and surveillance of bacterial infections and their antibiotic profiles are very critical for the creation of awareness, implementation of infection control measures, and proper management of such infections. This is essential in developing countries, especially in sub-Saharan Africa, where studies have shown that many health facilities have poor infection prevention and control (IPC) adherence. This challenge translates into increased bacterial infections caused by multi-drug-resistant bacteria, increasing morbidity and mortality [42,43]. For example, resistance to *β*-lactam antimicrobials among Enterobacteriaceae has been mainly caused by the acquisition of resistant genes that encodes for *β*-lactamase enzymes [44,45].

From this study, the frequent isolates from clinical specimens were *Escherichia coli* (40.9%), *Citrobacter* spp *(*32.6%), and *Klebsiella pneumoniae* (9.1%). This finding agrees with the report by Blomberg and colleagues from a study conducted in Tanzania, which indicated that *Escherichia coli*, *Klebsiella oxytoca*, and *Klebsiella pneumoniae* are common bacteria isolated from clinical samples of patients [46]. In addition, the findings of this study are consistent with a result in Ghana, which reported *Klebsiella pneumoniae*, *Enterobacter spp*., and *Escherichia coli* as the most isolated pathogenic Enterobacteriaceae from clinical specimens [47]. Again, the results of the isolated organisms conform to the findings from a study conducted by Feglo and colleagues in Ghana which reported that the most isolated Enterobacteriaceae from clinical specimens were predominantly *E*. *coli*, *Klebsiella pneumoniae*, and *Klebsiella oxytoca* [48]. These commonly isolated bacteria have developed resistance to widely used antibiotics across the globe, making it difficult to effectively treat infections caused by such bacteria [46]. Therefore, research must look into alternative drugs and interventions to minimize the effects of diseases from these bacteria.

Findings from this study have shown that *Citrobacter* spp *(*32.6%) is one of the common emerging uropathogens of urinary tract infections (UTIs). Out of the 75 isolates of *Citrobacter* spp collected, 58(35.8%) of the total number of *Citrobacter* spp. were isolated from urine specimens. In a study conducted by Sami and colleagues, the prevalence of *Citrobacter* spp was reported as 3.5% [49]. In addition, Deininger and colleagues reported a prevalence of 24.5% for *Citrobacter* spp [50]. As a common emerging uropathogens of UTIs, *Citrobacter* spp. has shown resistance to many antibiotics, such as penicillins, cephalosporins, aminoglycosides, and quinolones [51,52]. Furthermore, they are known to produce extended-spectrum beta-lactamases (ESBLs) and carbapenemases, which make them resistant to the most potent antibiotics; hence, they pose a threat to global public health especially due to the complications that are associated with them [51–53].

The persistence and recurrence of *Citrobacter* spp infections are attributed to factors such as antibiotic resistance, opportunistic nature, and biofilm formation [53]. Predisposing factors like urinary catheters or structural abnormalities allow them to establish infections in the urinary tract. With the ability to form biofilms, thus an encased protective matrix, *Citrobacter* spp can adhere to urinary tract surfaces, resist host immune responses, and increase antibiotic resistance. Hence, it is imperative to consider interventions such as strict infection control measures in hospitals, rational use of antibiotics, avoidance of unnecessary or prolonged use of catheters, targeted antibiotic therapy, surveillance and monitoring of antibiotic resistance patterns, and development of alternative treatments, to minimize or curb the impact of these infections.

The susceptibility profile of Enterobacteriaceae isolates in this study showed a low multidrug resistance (32.5%) compared to the 89.5% reported by Agyepong and colleagues [4]. This study has shown a relatively low resistance toward ampicillin (74.4%), cefuroxime (58.3%), and cefotaxime (54.8%) as compared to the report by Agyepong and colleagues, which indicated a high resistance of Gram-negative bacteria towards, ampicillin (94.4%), cefuroxime (79.0%) and cefotaxime (71.3%) [4]. Similarly, the results from this study were contrary to the study by Feglo and colleagues, which reported isolates resistant to ampicillin (91.7%) and cefuroxime (70.6%) [48]. Resistance of isolates towards cefotaxime (54.8%) was high in this study compared to the 48.1% reported by Feglo and colleagues [48]. Results from the present study also support the findings by Labi and colleagues, which indicated high antibiotic resistance among members of the Enterobacteriaceae at the Korle Bu Teaching Hospital to the combination of ampicillin/ gentamicin and ampicillin/ cefotaxime [54]. The resistance among Enterobacteriaceae to ampicillin and other commonly used antibiotics is consistent with other studies in sub-Saharan African countries such as Nigeria [55], Rwanda [56], Ethiopia [57], Zimbabwe [58], and Tanzania [59]. The trend of antibiotic resistance rates in the sub-region could be due to high antibiotic selection pressure due to the unregulated availability of antibiotics over the counter and cheap substandard antibiotics influx in the sub-Saharan region [14,60–62]. This outcome has compromised the choice of antibiotics available for treatment, leading to higher-class antibiotics as the preference. The high cost of higher-class antibiotics makes their applicability difficult due to the economic hardships in the region; hence, morbidity and mortality rates keep increasing. By strengthening public education on antibiotic stewardship in the community and strictly adhering to the use and prescription of antibiotics, I believe the impact of this situation will be minimized.

ESBL production by Gram-negative bacteria poses a great challenge in the management of Gram-negative bacterial infections. ESBL production may be associated with MDR. From the study, the prevalence of ESBL producers among Enterobacteriaceae was 49.1% (113/230), with the most ESBL-producing organism being *E*. *coli* (57.5%). The prevalence (49.1%) of the ESBL was lower than that reported by Feglo and colleagues in a study conducted in Ghana which reported a prevalence of 57.8% [48]. Furthermore, findings from this study showed that ESBL prevalence was higher than the ESBL prevalence (37.96%) reported by Oduro-Mensah and colleagues in Ghana [63]. The difference in the prevalence could be attributed to the difference in geographical location, sample size, and sampling period. Also, the result on the ESBL producers indicated that the ESBL harbouring isolates were predominantly from outpatients (76.1%). However, in-patients are expected to harbour more ESBL producers due to their exposure and risk to nosocomial infections and more antibiotics usage. For instance, the study by Khanfar and colleagues in Saudi Arabia reported ESBL producers to be higher in in-patients (15.4%) than outpatients (4.5%) [64]. In like manner, Ouedraogo and colleagues in Burkina Faso also reported ESBL prevalence to be higher in hospitalized patients [65]. The high prevalence of ESBL producers among outpatients in this study is quite alarming, and this could be due to the possible self-medication resulting from the unregulated availability of antibiotics over the counter. The outcome of this situation may have escalated the transmission of the plasmid-encoded genes within the community; hence, the community may be serving as a reservoir for ESBL and requires immediate attention to prevent the possible outbreak of ESBL-carrying strains of Enterobacteriaceae.

Carbapenem antibiotics are considered last-resort drugs for the treatment of severe bacterial infections, thus, infections caused by bacteria that are resistant to other antibiotics [66]. The presence of the genes *blaOXA-48* and *blaNDM-1* in *Proteus* spp, *Citrobacter* spp, and *E*. *coli* resistant strains suggests the presence of resistance mechanisms that can confer resistance to carbapenem antibiotics. This shows that strains carrying these genes are resistant to carbapenems, limiting their effectiveness. Carbapenem-resistant strains are associated with higher mortality rates and treatment failure; hence, the presence of these genes in the study population suggests a higher likelihood of treatment failure when carbapenems are used as a treatment option [66,67]. Furthermore, their presence in multiple bacterial species increases the risk of their dissemination within the study population and potentially beyond. The ability of these resistance genes to be transferred horizontally between bacteria poses a significant challenge in controlling their spread. The outcome of studies in Ghana conducted by Codjoe and colleagues [37] and Quansah and colleagues [35] indicated the presence of both *blaNDM-1* and *blaOXA-48* in the study population. In addition, surveillance studies in the Western world and developed countries such as Germany [68] and China [69] have indicated several rates of carbapenemase producers in these countries.

Globally, a broader view must be given to this alarming situation of AMR, especially because possible importation of resistance genes has been found in a few countries, such as South Africa, due to tourism and migration [70]. Research must consider the development of rapid diagnostic techniques to serve as screening tools for travelers who may be carrying multidrug-resistant genes to avert possible spread across countries.

## Conclusion

The study has demonstrated the high prevalence of carbapenem-resistant and high ESBL production among Enterobacteriaceae. ESBL production was found among 49.1% of bacterial isolates, and the prevalence of MDR and CRE were 35.2% and 5.7%, respectively. *E*. *coli*, *Citrobacter* spp., *K*. *pneumoniae*, and *Klebsiella oxytoca* were the most common causative organisms isolated. The study found high rates of MDR among *E*. *coli* and *Citrobacter* spp. The Enterobacteriaceae were most sensitive towards amikacin, imipenem, and meropenem and most resistant towards ampicillin, cefuroxime, and cefotaxime. Also, the presence of the *blaNDM-1* gene and *blaOXA-48* gene were detected for 11 and 13 CRE isolates, respectively, via PCR. These results should guide the empirical treatment of bacterial infections caused by Enterobacteriaceae in Cape Coast Teaching Hospital of Ghana. Other recommendations may include PCR to determine the types of ESBL in CCTH and adopting strict infection control measures to prevent the rapid spread of resistance.

## Supporting information

S1 Data (XLSX)

## References

[1] World Health Organization. Antimicrobial resistance:: Global report on surveillance; 2014.

[2] World Health Organization 2018 Global Antimicrobial Resistance Surveillance System (GLASS) Report Early Implementation 2017–2018 2018.

[3] Singh N, Manchanda V. Control of multidrug-resistant Gram-negative bacteria in low- and middle-income countries-high impact interventions without much resources. Clin Microbiol Infect. 2017;23:216–8. doi: 10.1016/j.cmi.2017.02.034 28274769

[4] Agyepong N, Govinden U, Owusu-Ofori A, Essack SY. Multidrug-resistant gram-negative bacterial infections in a teaching hospital in Ghana. Antimicrob Resist Infect Control. 2018;7:37. doi: 10.1186/s13756-018-0324-2 29541448 PMC5845144

[5] Méndez-Vilas A. Microbial pathogens and strategies for combating them: Science, technology and education 2013;4. Badajoz: Formatex Research Center.

[6] Rossolini GM, Mantengoli E, Docquier J-D, Musmanno RA, Coratza G. Epidemiology of infections caused by multiresistant gram-negatives: ESBLs, MBLs, panresistant strains. J Nat Sci. 2007;30:332–9. 17802921

[7] Nordmann P, Naas T, Poirel L. Global spread of Carbapenemase-producing Enterobacteriaceae. Emerging Infectious Diseases. 2011;17:1791–8. doi: 10.3201/eid1710.110655 22000347 PMC3310682

[8] Paterson DL. Resistance in gram-negative bacteria: Enterobacteriaceae. American Journal of Infection Control. 2006;34:S20–8; discussion S64-73. doi: 10.1016/j.ajic.2006.05.238 16813978

[9] Bitew A, Tsige E. High Prevalence of Multidrug-Resistant and Extended-Spectrum β-Lactamase-Producing Enterobacteriaceae: A Cross-Sectional Study at Arsho Advanced Medical Laboratory, Addis Ababa, Ethiopia. Journal of Tropical Medicine. 2020;2020:6167234. doi: 10.1155/2020/6167234 32411256 PMC7210541

[10] Shilpakar A, Ansari M, Rai KR, Rai G, Rai SK. Prevalence of multidrug-resistant and extended-spectrum beta-lactamase producing Gram-negative isolates from clinical samples in a tertiary care hospital of Nepal. Trop Med Health. 2021;49:23. doi: 10.1186/s41182-021-00313-3 33691795 PMC7948344

[11] Bradford PA. Extended-spectrum beta-lactamases in the 21st century: characterization, epidemiology, and detection of this important resistance threat. Clin Microbiol Rev. 2001;14:933–51, table of contents. doi: 10.1128/CMR.14.4.933-951.2001 11585791 PMC89009

[12] Ben-Ami R, Rodríguez-Baño J, Arslan H, Pitout JDD, Quentin C, Calbo ES, et al. A multinational survey of risk factors for infection with extended-spectrum beta-lactamase-producing enterobacteriaceae in nonhospitalized patients. Clin Infect Dis. 2009;49:682–90. doi: 10.1086/604713 19622043

[13] Perez F, van Duin D. Carbapenem-resistant Enterobacteriaceae: a menace to our most vulnerable patients. Cleveland Clinic Journal of Medicine. 2013;80:225–33. doi: 10.3949/ccjm.80a.12182 23547093 PMC3960994

[14] Mushi MF, Mshana SE, Imirzalioglu C, Bwanga F. Carbapenemase genes among multidrug resistant gram negative clinical isolates from a tertiary hospital in Mwanza, Tanzania. BioMed Research International. 2014;2014:303104. doi: 10.1155/2014/303104 24707481 PMC3953670

[15] Tada T, Miyoshi-Akiyama T, Dahal RK, Mishra SK, Ohara H, Shimada K, et al. Dissemination of multidrug-resistant Klebsiella pneumoniae clinical isolates with various combinations of carbapenemases (NDM-1 and OXA-72) and 16S rRNA methylases (ArmA, RmtC and RmtF) in Nepal. International Journal of Antimicrobial Agents. 2013;42:372–4. doi: 10.1016/j.ijantimicag.2013.06.014 23978353

[16] Leski TA, Taitt CR, Bangura U, Stockelman MG, Ansumana R, Cooper WH, et al. High prevalence of multidrug resistant Enterobacteriaceae isolated from outpatient urine samples but not the hospital environment in Bo, Sierra Leone. BMC Infectious Diseases. 2016;16:167. doi: 10.1186/s12879-016-1495-1 27090787 PMC4836052

[17] Leski T, Vora GJ, Taitt CR. Multidrug resistance determinants from NDM-1-producing Klebsiella pneumoniae in the USA. International Journal of Antimicrobial Agents. 2012;40:282–4. doi: 10.1016/j.ijantimicag.2012.05.019 22817914

[18] Papp-Wallace KM, Endimiani A, Taracila MA, Bonomo RA. Carbapenems: past, present, and future. Antimicrobial Agents and Chemotherapy. 2011;55:4943–60. doi: 10.1128/AAC.00296-11 21859938 PMC3195018

[19] Kula B, Djordjevic G, Robinson JL. A systematic review: can one prescribe carbapenems to patients with IgE-mediated allergy to penicillins or cephalosporins? Clin Infect Dis. 2014;59:1113–22. doi: 10.1093/cid/ciu587 25048853

[20] Torok ME, Moran E, Cooke FJ. Oxford Handbook of Infectious Diseases and Microbiology. In: Oxford handbook of infectious diseases and microbiology. 2nd ed. [Oxford]: Oxford University Press; 2016.

[21] Wilson H, Török ME. Extended-spectrum β-lactamase-producing and carbapenemase-producing Enterobacteriaceae. Microbial Genomics 2018. doi: 10.1099/mgen.0.000197 30265236 PMC6202456

[22] Ssekatawa K, Byarugaba DK, Wampande E, Ejobi F. A systematic review: the current status of carbapenem resistance in East Africa. BMC Res Notes. 2018;11:629. doi: 10.1186/s13104-018-3738-2 30170613 PMC6119249

[23] Moyo SJ, Aboud S, Kasubi M, Lyamuya EF, Maselle SY. Antimicrobial resistance among producers and non-producers of extended spectrum beta-lactamases in urinary isolates at a tertiary Hospital in Tanzania. BMC Research Notes. 2010;3:348. doi: 10.1186/1756-0500-3-348 21184671 PMC3017072

[24] Warnes SL, Highmore CJ, Keevil CW. Horizontal transfer of antibiotic resistance genes on abiotic touch surfaces: implications for public health. Am Soc Microbiol. 2012;3:6–12. Am Soc Microbiol. 2012;3:6. doi: 10.1128/mBio.00489-12 23188508 PMC3509412

[25] Moyo S, Haldorsen B, Aboud S, Blomberg B, Maselle SY, Sundsfjord A, et al. Identification of VIM-2-producing Pseudomonas aeruginosa from Tanzania is associated with sequence types 244 and 640 and the location of blaVIM-2 in a TniC integron. Antimicrobial Agents and Chemotherapy. 2015;59:682–5. doi: 10.1128/AAC.01436-13 25331700 PMC4291420

[26] Poirel L, Revathi G, Bernabeu S, Nordmann P. Detection of NDM-1-producing Klebsiella pneumoniae in Kenya. Antimicrobial Agents and Chemotherapy. 2011;55:934–6. doi: 10.1128/AAC.01247-10 21115785 PMC3028766

[27] Revathi G, Siu LK, Lu P-L, Huang L-Y. First report of NDM-1-producing Acinetobacter baumannii in East Africa. Int J Infect Dis. 2013;17:e1255–8. doi: 10.1016/j.ijid.2013.07.016 24176550

[28] Perovic O, Ismail H, Quan V, Bamford C, Nana T, Chibabhai V, et al. Carbapenem-resistant Enterobacteriaceae in patients with bacteraemia at tertiary hospitals in South Africa, 2015 to 2018. European Journal of Clinical Microbiology & Infectious Diseases. 2020;39:1287–94. doi: 10.1007/s10096-020-03845-4 32124106

[29] Adesanya OA, Igwe HA. Carbapenem-resistant Enterobacteriaceae (CRE) and gram-negative bacterial infections in south-west Nigeria: a retrospective epidemiological surveillance study. AIMS Public Health. 2020;7:804–15. doi: 10.3934/publichealth.2020062 33294483 PMC7719558

[30] Vasaikar SD, Hanise P, Abaver DT. Epidemiology, risk factors and molecular analysis of carbapenem-resistant Enterobacteriaceae (CRE) in Mthatha, Eastern Cape, South Africa. International Journal of Infectious Diseases. 2020;101:54. doi: 10.1016/j.ijid.2020.09.173

[31] Thomas TSM, Duse AG. Epidemiology of carbapenem-resistant Enterobacteriaceae (CRE) and comparison of the phenotypic versus genotypic screening tests for the detection of carbapenemases at a tertiary level, academic hospital in Johannesburg, South Africa. Southern African Journal of Infectious Diseases. 2018:1–7. doi: 10.1080/23120053.2018.1509184

[32] Brink Adrian, Coetzee Jennifer, Clay Cornelis, Corcoran Craig, Johan van Greu, Deetlefs J D, et al. The spread of carbapenem-resistant Enterobacteriaceae in South Africa: Risk factors for acquisition and prevention. South African Medical Journal. 2012;102:599–601. doi: 10.7196/samj.5789 22748433

[33] Ngbede EO, Adekanmbi F, Poudel A, Kalalah A, Kelly P, Yang Y, et al. Concurrent Resistance to Carbapenem and Colistin Among Enterobacteriaceae Recovered From Human and Animal Sources in Nigeria Is Associated With Multiple Genetic Mechanisms. Front. Microbiol. 2021;12:740348. doi: 10.3389/fmicb.2021.740348 34690985 PMC8528161

[34] El Wartiti MA, Bahmani F-Z, Elouennass M, Benouda A. Prevalence of carbapenemase-producing enterobacteriaceae in a University Hospital in Rabat, Morocco: A 19-months prospective study. International Arabic Journal of Antimicrobial Agents. 2012;2:1–6. doi: 10.3823/718

[35] Quansah E, Amoah Barnie P, Omane Acheampong D, Obiri-Yeboah D, Odarkor Mills R, Asmah E, et al. Geographical Distribution of β-Lactam Resistance among Klebsiella spp. from Selected Health Facilities in Ghana. Tropical Medicine and Infectious Disease 2019. doi: 10.3390/tropicalmed4030117 31484298 PMC6789473

[36] Oduro P. Prevalence of Carbapenemase Producing Enterobacteriaceae in Urinary Tract Infection Patients in Ghana. undefined. 2016.

[37] Codjoe FS, Donkor ES, Smith TJ, Miller K. Phenotypic and Genotypic Characterization of Carbapenem-Resistant Gram-Negative Bacilli Pathogens from Hospitals in Ghana. Microbial Drug Resistance. 2019;25:1449–57. doi: 10.1089/mdr.2018.0278 31237486

[38] Cape Coast Teaching Hospital. Cape Coast Teaching Hospital:: 2018 Performance Report. Cape Coast; 2018.

[39] Ghana Statistical Service. 2010 Population & Housing Census:: District analytical report. Cape Coast Municipality. Accra; 2010.

[40] CLSI. Performance Standards for Antimicrobial Susceptibility Testing. 28th ed. Wayne, PA; 2018.

[41] Poirel L, Walsh TR, Cuvillier V, Nordmann P. Multiplex PCR for detection of acquired carbapenemase genes. Diagnostic Microbiology and Infectious Disease. 2011;70:119–23. doi: 10.1016/j.diagmicrobio.2010.12.002 21398074

[42] Huttner A, Harbarth S, Carlet J, Cosgrove S, Goossens H, Holmes A, et al. Antimicrobial resistance: a global view from the 2013 World Healthcare-Associated Infections Forum. Antimicrob Resist Infect Control. 2013;2:31. doi: 10.1186/2047-2994-2-31 24237856 PMC4131211

[43] Samuel S, Kayode O, Musa O, Nwigwe G, Aboderin A, Salami T, et al. Nosocomial infections and the challenges of control in developing countries. Afr J Clin Exp Microbiol. 2010;11:102–9.

[44] Shaikh S, Fatima J, Shakil S, Rizvi SMD, Kamal MA. Antibiotic resistance and extended spectrum beta-lactamases: Types, epidemiology and treatment. Saudi Journal of Biological Sciences. 2015;22:90–101. doi: 10.1016/j.sjbs.2014.08.002 25561890 PMC4281622

[45] Jacoby GA, Munoz-Price LS. The new beta-lactamases. N Engl J Med. 2005;352:380–91. doi: 10.1056/NEJMra041359 15673804

[46] Blomberg B, Jureen R, Manji KP, Tamim BS, Mwakagile DSM, Urassa WK, et al. High rate of fatal cases of pediatric septicemia caused by gram-negative bacteria with extended-spectrum beta-lactamases in Dar es Salaam, Tanzania. Journal of Clinical Microbiology. 2005;43:745–9. doi: 10.1128/JCM.43.2.745-749.2005 15695674 PMC548071

[47] Codjoe FS. Detection and characterisation of carbapenem-resistant gram-negative bacilli infections in Ghana. 10.1089/mdr.2018.0278 31237486

[48] Feglo PK, Gbedema SY, Quay SNA, Adu-Sarkodie Y, Opoku-Okrah C. Occurrence, species distribution and antibiotic resistance of Proteus isolates: A case study at the Komfo Anokye Teaching Hospital (KATH) in Ghana. International Journal of Pharma Sciences and Research. 2010;1.

[49] Sami H, Sultan A, Rizvi M, Khan F, Ahmad S, Shukla I, et al. Citrobacter as a uropathogen, its prevalence and antibiotics susceptibility pattern. CHRISMED J Health Res. 2017;4:23. doi: 10.4103/2348-3334.196037

[50] Deininger S, Gründler T, Deininger SHM, Lütcke K, Lütcke H, Agbesi J, et al. The Antimicrobial Resistance (AMR) Rates of Uropathogens in a Rural Western African Area-A Retrospective Single-Center Study from Kpando, Ghana. Antibiotics. 2022;11:1808. doi: 10.3390/antibiotics11121808 36551465 PMC9774093

[51] Ranjan KP, Ranjan N. Citrobacter: An emerging health care associated urinary pathogen. Urology Annals. 2013;5:313–4. 24311922 PMC3836000

[52] Metri BC, Jyothi P, Peerapur BV. Antibiotic resistance in Citrobacter spp. isolated from urinary tract infection. Urology Annals. 2013;5:312–3. doi: 10.4103/0974-7796.120295 24311921 PMC3835999

[53] Flores-Mireles AL, Walker JN, Caparon M, Hultgren SJ. Urinary tract infections: epidemiology, mechanisms of infection and treatment options. Nature Reviews Microbiology. 2015;13:269–84. doi: 10.1038/nrmicro3432 25853778 PMC4457377

[54] Labi A-K, Obeng-Nkrumah N, Bjerrum S, Enweronu-Laryea C, Newman MJ. Neonatal bloodstream infections in a Ghanaian Tertiary Hospital: Are the current antibiotic recommendations adequate? BMC Infect Dis. 2016;16:598. doi: 10.1186/s12879-016-1913-4 27776490 PMC5078915

[55] Ogbolu DO, Daini OA, Ogunledun A, Alli AO, Webber MA. High levels of multidrug resistance in clinical isolates of Gram-negative pathogens from Nigeria. International Journal of Antimicrobial Agents. 2011;37:62–6. doi: 10.1016/j.ijantimicag.2010.08.019 21074376

[56] Carroll M, Rangaiahagari A, Musabeyezu E, Singer D, Ogbuagu O. Five-Year Antimicrobial Susceptibility Trends Among Bacterial Isolates from a Tertiary Health-Care Facility in Kigali, Rwanda. The American Journal of Tropical Medicine and Hygiene. 2016;95:1277–83. doi: 10.4269/ajtmh.16-0392 27799637 PMC5154439

[57] Muluye D, Wondimeneh Y, Ferede G, Nega T, Adane K, Biadgo B, et al. Bacterial isolates and their antibiotic susceptibility patterns among patients with pus and/or wound discharge at Gondar university hospital. BMC Research Notes. 2014;7:619. doi: 10.1186/1756-0500-7-619 25201246 PMC4167130

[58] Mbanga J, Dube S, Munyanduki H. Prevalence and drug resistance in bacteria of the urinary tract infections in Bulawayo province, Zimbabwe. East Afr J Public Health. 2010;7:229–32. doi: 10.4314/eajph.v7i3.64733 21516960

[59] Kumburu HH, Sonda T, Mmbaga BT, Alifrangis M, Lund O, Kibiki G, et al. Patterns of infections, aetiological agents and antimicrobial resistance at a tertiary care hospital in northern Tanzania. Tropical Medicine and International Health. 2017;22:454–64. doi: 10.1111/tmi.12836 28072493

[60] Mshana SE, Matee M, Rweyemamu M. Antimicrobial resistance in human and animal pathogens in Zambia, Democratic Republic of Congo, Mozambique and Tanzania: an urgent need of a sustainable surveillance system. Annals of Clinical Microbiology and Antimicrobials. 2013;12:28. doi: 10.1186/1476-0711-12-28 24119299 PMC3852305

[61] Ocan M, Bwanga F, Bbosa GS, Bagenda D, Waako P, Ogwal-Okeng J, et al. Patterns and predictors of self-medication in northern Uganda. PLoS ONE. 2014;9:e92323. doi: 10.1371/journal.pone.0092323 24658124 PMC3962384

[62] Donkor ES, Tetteh-Quarcoo PB, Nartey P, Agyeman IO. Self-medication practices with antibiotics among tertiary level students in Accra, Ghana: a cross-sectional study. International Journal of Environmental Research and Public Health. 2012;9:3519–29. doi: 10.3390/ijerph9103519 23202760 PMC3509469

[63] Oduro-Mensah D, Obeng-Nkrumah N, Bonney EY, Oduro-Mensah E, Twum-Danso K, Osei YD, et al. Genetic characterization of TEM-type ESBL-associated antibacterial resistance in Enterobacteriaceae in a tertiary hospital in Ghana. Annals of Clinical Microbiology and Antimicrobials. 2016;15:29. doi: 10.1186/s12941-016-0144-2 27145868 PMC4857374

[64] Khanfar HS, Bindayna KM, Senok AC, Botta GA. Extended spectrum beta-lactamases (ESBL) in Escherichia coli and Klebsiella pneumoniae: trends in the hospital and community settings. J Infect Dev Ctries. 2009;3:295–9. doi: 10.3855/jidc.127 19759493

[65] Ouedraogo A-S, Sanou M, Kissou A, Sanou S, Solaré H, Kaboré F, et al. High prevalence of extended-spectrum ß-lactamase producing enterobacteriaceae among clinical isolates in Burkina Faso. BMC Infectious Diseases. 2016;16:326. doi: 10.1186/s12879-016-1655-3 27400864 PMC4939587

[66] Karbasizade Vajihe, reyhaneh Jafari Sharareh Moghim, Jahani Somaieh, Maryam Mohammadi Sichani. The Frequency of blaOXA-48 and blaNDM-1 Genes in the Enterobacteriaceae Strains Isolated from Clinical Samples. Archives of Pharmacy Practice. 2020;11:163–8.

[67] Székely E, Damjanova I, Jánvári L, Vas KE, Molnár S, Bilca DV, et al. First description of bla(NDM-1), bla(OXA-48), bla(OXA-181) producing Enterobacteriaceae strains in Romania. International journal of medical microbiology: IJMM. 2013;303:697–700. doi: 10.1016/j.ijmm.2013.10.001 24183483

[68] Ehrhard I, Karaalp A-K, Hackel T, Höll G, Rodewald N, Reif U, et al. Prävalenzerhebung zum Vorkommen von Carbapenemase-Bildnern in sächsischen Kliniken. [Prevalence of carbapenemase-producing bacteria in hospitals in Saxony, Germany]. Bundesgesundheitsblatt—Gesundheitsforschung—Gesundheitsschutz. 2014;57:406–13. doi: 10.1007/s00103-013-1914-z 24658670

[69] Qin S, Fu Y, Zhang Q, Qi H, Wen JG, Xu H, et al. High incidence and endemic spread of NDM-1-positive Enterobacteriaceae in Henan Province, China. Antimicrobial Agents and Chemotherapy. 2014;58:4275–82. doi: 10.1128/AAC.02813-13 24777095 PMC4136005

[70] Brink A, Coetzee J, Clay C, Corcoran C, van Greune J, Deetlefs JD, et al. The spread of carbapenem-resistant Enterobacteriaceae in South Africa: Risk factors for acquisition and prevention. South African Medical Journal. 2012;102:599–601. doi: 10.7196/samj.5789 22748433

